# The steroid metabolome in women with premenstrual dysphoric disorder during GnRH agonist-induced ovarian suppression: effects of estradiol and progesterone addback

**DOI:** 10.1038/tp.2017.146

**Published:** 2017-08-08

**Authors:** T V Nguyen, J M Reuter, N W Gaikwad, D M Rotroff, H R Kucera, A Motsinger-Reif, C P Smith, L K Nieman, D R Rubinow, R Kaddurah-Daouk, P J Schmidt

**Affiliations:** 1 Behavioral Endocrinology Branch, NIMH IRP/NIH/HHS, Bethesda, MD, USA; 2 Department of Psychiatry and Obstetrics-Gynecology, McGill University Health Center, Montreal, QC, Canada; 3 Department of Nutrition and Environmental Toxicology, West Coast Metabolomics Center, University of California, Davis, Davis, CA, USA; 4 Department of Biostatistics, Bioinformatics Research Center, North Carolina State University, Raleigh, NC, USA; 5 Department of Statistics, North Carolina State University, Raleigh, NC, USA; 6 Diabetes, Endocrine and Obesity Branch, NIDDK, NIH, DHSS, Bethesda, MD, USA; 7 Department of Psychiatry, University of North Carolina School of Medicine, Chapel Hill, NC, USA; 8 Department of Psychiatry, Duke University Medical Center, Durham, NC, USA; 9 Duke Institute for Brain Sciences, Duke University, Durham, NC, USA

## Abstract

Clinical evidence suggests that symptoms in premenstrual dysphoric disorder (PMDD) reflect abnormal responsivity to ovarian steroids. This differential steroid sensitivity could be underpinned by abnormal processing of the steroid signal. We used a pharmacometabolomics approach in women with prospectively confirmed PMDD (*n*=15) and controls without menstrual cycle-related affective symptoms (*n*=15). All were medication-free with normal menstrual cycle lengths. Notably, women with PMDD were required to show hormone sensitivity in an ovarian suppression protocol. Ovarian suppression was induced for 6 months with gonadotropin-releasing hormone (GnRH)-agonist (Lupron); after 3 months all were randomized to 4 weeks of estradiol (E2) or progesterone (P4). After a 2-week washout, a crossover was performed. Liquid chromatography/tandem mass spectrometry measured 49 steroid metabolites in serum. Values were excluded if >40% were below the limit of detectability (*n*=21). Analyses were performed with Wilcoxon rank-sum tests using false-discovery rate (*q*<0.2) for multiple comparisons. PMDD and controls had similar basal levels of metabolites during Lupron and P4-derived neurosteroids during Lupron or E2/P4 conditions. Both groups had significant increases in several steroid metabolites compared with the Lupron alone condition after treatment with E2 (that is, estrone-SO_4_ (*q*=0.039 and *q*=0.002, respectively) and estradiol-3-SO_4_ (*q*=0.166 and *q*=0.001, respectively)) and after treatment with P4 (that is, allopregnanolone (*q*=0.001 for both PMDD and controls), pregnanediol (*q*=0.077 and *q*=0.030, respectively) and cortexone (*q*=0.118 and *q*=0.157, respectively). Only sulfated steroid metabolites showed significant diagnosis-related differences. During Lupron plus E2 treatment, women with PMDD had a significantly attenuated increase in E2-3-sulfate (*q*=0.035) compared with control women, and during Lupron plus P4 treatment a decrease in DHEA-sulfate (*q*=0.07) compared with an increase in controls. Significant effects of E2 addback compared with Lupron were observed in women with PMDD who had significant decreases in DHEA-sulfate (*q*=0.065) and pregnenolone sulfate (*q*=0.076), whereas controls had nonsignificant increases (however, these differences did not meet statistical significance for a between diagnosis effect). Alterations of sulfotransferase activity could contribute to the differential steroid sensitivity in PMDD. Importantly, no differences in the formation of P4-derived neurosteroids were observed in this otherwise highly selected sample of women studied under controlled hormone exposures.

## Introduction

Premenstrual dysphoric disorder (PMDD) is a clinically distinct affective disorder characterized by recurrent and distressing mood and behavioral symptoms during the luteal phase of the menstrual cycle that remit within a few days from the onset of menses.^1, 2, 3^ Levels of circulating ovarian steroids, estradiol (E2) and progesterone (P4) and hypothalamic–pituitary–ovarian axis function are normal.^4^ However, when ovarian steroid secretion is suppressed by gonadotropin-releasing hormone (GnRH) receptor agonists, women with PMDD experience remission of symptoms, which recur when physiologic doses of E2/P4 are added back. In contrast, asymptomatic controls undergoing identical hormone manipulations experience no changes in mood.^5^ Thus, in women with PMDD, clinical evidence suggests that symptoms are triggered by a differential central nervous system (CNS) response to physiologically normal changes in E2/P4. Furthermore, imaging studies identified brain regions modulated by E2/P4 (or their neuroactive metabolites), some of which respond differently in women with PMDD despite similar exposures to E2/P4, including the amygdala, striatum, medial orbitofrontal cortex, anterior cingulate cortex and prefrontal cortex.^6, 7, 8, 9, 10, 11, 12, 13, 14, 15^ One candidate hormone that could mediate the differential regulation of brain circuitry in PMDD, and the triggering of symptoms by at least progesterone, is the ring A-reduced neurosteroid allopregnanolone. Despite the absence of consistent demonstrations of abnormal peripheral levels of allopregnanolone in PMDD compared with control women,^16, 17, 18, 19, 20, 21, 22^ there is preliminary evidence to support the hypothesis that changes in the production of or alterations in the metabolism of allopregnanolone from progesterone could serve as an 'affective switch' in susceptible women (reviewed in Schiller.^16, 23^ These observations not only suggest that the symptoms of PMDD are accompanied by ovarian steroid-related alterations in the activity of neuronal circuits underlying reward, social cognition and affective states,^4^ but also that ovarian steroids could be processed differently in women with PMDD in peripheral tissues and potentially in the CNS (or alternatively reflect differential signal transduction within the CNS).

Abnormalities in steroid metabolism are present in several conditions, including prostate cancer, breast cancer and polycystic ovary syndrome,^24, 25, 26, 27^ and metabolomics platforms have been employed to investigate biochemical systems (for example, lipids and neurotransmitter metabolites) in metabolic and psychiatric conditions.^28, 29, 30^

We hypothesized that women with PMDD would display altered steroid metabolic profiles/signatures compared with asymptomatic control women during controlled and standardized exposures to ovarian steroids. To test this, we used a steroid-based metabolomics platform to compare asymptomatic controls and women with PMDD in whom symptom remission and recurrence (that is, E2/P4 sensitivity) were confirmed by ovarian steroid manipulation.^5^ The main goal of this study was to examine the potential differences in the metabolic processing of standardized doses of ovarian steroids in women with PMDD and controls. Metabolomics tools enable identification and quantification of tens to thousands of compounds that represent changes in biochemical pathways^31, 32^ in response to treatment. Metabolomics strategies have also mapped global biochemical changes in depression, characterized the effects of selective serotonergic re-uptake inhibitors on metabolic pathways and defined several pathways implicated in individual variation in response to these medications.^33, 34, 35^

## Materials and methods

### Participant selection

We studied 15 women with PMDD aged 23–48 years. All were medication-free, with regular menstrual cycles (range, 21–35 days), not medically ill and not pregnant. Women with PMDD were self-referred in response to newspaper advertisements or were referred by their physician. Before study entry PMDD was confirmed in these women based on criteria outlined in the American Psychiatric Association’s Diagnostic and Statistical Manual of Mental Disorders, fourth edition (DSM-IV).^36^ Women completed 3 months of prospective daily ratings using a four-item 100-mm visual-analog scale that confirmed the timing and severity of their self-reported menstrually related mood symptoms (irritability, sadness, anxiety and mood swings) as described previously.^5, 37^ The mean score of at least one negative mood symptoms was at least 30% higher (relative to the range of the scale used by each woman) in the week before menstruation compared with the week after the cessation of menstruation in at least two of the three cycles. Functional impairment was characterized as a daily rating form (DRF)^38^ score of 2 (minimal) or higher on one of 4 questions related to functional impairment (that is, stayed at home or avoided social activities, had conflicts or problems with people, symptoms interfered with relationships at work or home or symptoms interfered with work productivity) in at least 3 days out of 7 days pre-menses. Finally, DRF ratings and the results of both a semi-structured interview and a self-report questionnaire (that is, the Menstrual Screening Questionnaire and the Menstrual Assessment Form, respectively) confirmed that all women met the required number of symptoms specified in the DSM. Women with significant negative mood symptoms (on the DRF) during the follicular phase of the menstrual cycle were excluded. Thus, the diagnostic criteria for PMDD were augmented by the severity criterion of a 30% or greater increase in the mean negative mood during the week before menses compared with the week after menses—a more stringent criterion than that of DSM-IV or V.^1, 36^

We also recruited a group of 15 control women, none of whom had premenstrual mood symptoms using the same daily rating scales during a 2-month baseline period.

The women with PMDD had no current Axis I psychiatric diagnosis within the past 2 years per Structured Clinical Interview for DSM-IV (SCID),^39^ while controls had neither current nor past Axis I diagnoses (also confirmed by SCID).

This study was approved by the Central Neuroscience Institutional Review Board within the NIMH IRP. All women provided written informed consent, and all received payment for participation according to the NIH intramural guidelines.

### Hormone manipulation protocol

**Figure 1:**
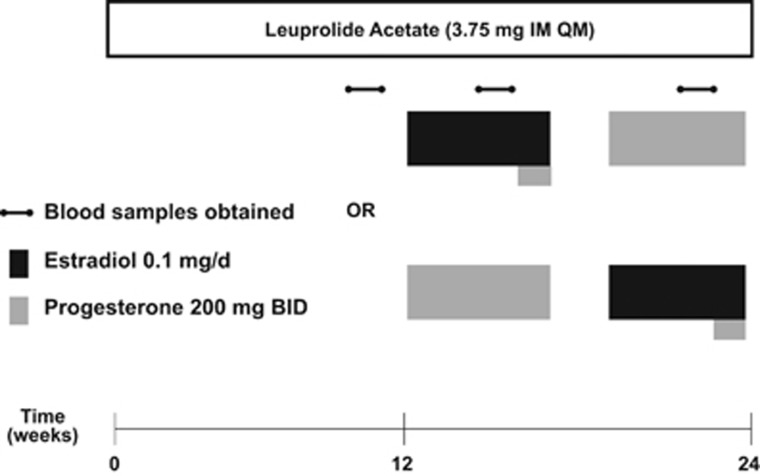
Protocol schematic for clinical hormone suppression/addback protocol. Between 2 and 5 days after onset of menses, women with PMDD and control women received six monthly intramuscular injections of 3.75-mg leuprolide (Lupron), which after an initial stimulation suppresses ovarian function. Clinic visits occurred every 2 weeks. Plasma FSH, LH, estradiol and progesterone levels were measured at each visit to confirm ovarian suppression. Following 3 months of leuprolide alone, all participants (while continuing to receive monthly leuprolide injections for another 3 months) were randomly assigned in a double-blind, crossover manner to receive either E2 (100 mg daily by skin patch) or P4 (200 mg vaginal suppository twice daily) replacement lasting for 5 weeks each (with a 2-week washout between hormone administration periods). All women used a patch and a suppository each day during the hormonal addback to maintain the patency of the double-blind. After 4 weeks of E2 all women received 1 week of P4 to induce menses (at the end of a 4-week E2 exposure). Significant recurrence of PMDD symptoms was defined by a weekly average DRF score of greater than three (moderate severity) in irritability, anxiety or sadness.^40, 41^ Controls were defined by an absence of affective symptoms throughout the 6-month hormone-manipulation protocol (that is, no weekly average score >2). DRF, daily rating form; E2, estradiol; P4, progesterone; PMDD, premenstrual dysphoric disorder.

Participants received six monthly injections of the GnRH agonist leuprolide acetate, Lupron (3.75 mg intramuscularly). Plasma follicle stimulating hormone (FSH), luteinizing hormone (LH), estradiol and progesterone levels were measured at study visits every 2 weeks to confirm adequate gonadal suppression. Following 3 months of Lupron alone, participants were randomly assigned in a double-blind, crossover manner to receive either E2 (100 mcg daily by skin patch, Noven Pharmaceuticals Jersey City, NJ, USA, Watson Pharmaceuticals, Parsippany, NJ, USA) or P4 (200 mg vaginal suppository twice daily, Upsher-Smith Laboratories; NIH Pharmacy, Bethesda, MD, USA) replacement lasting for 5 weeks (with a 2-week washout between hormone administration periods) while continuing to receive Lupron injections (Figure 1). All women used a patch and a suppository each day during the hormonal addback to maintain the patency of the double-blind. This standardized the exposures to physiologic levels of ovarian steroids to avoid the confound of varying levels of estradiol and progesterone across the normal menstrual cycle. All women used the appropriate placebo patch or suppository daily during the 12-week addback.

### Symptom ratings and criteria for response

Women completed a modified DRF,^38^ daily to measure symptom severity during the 6-month hormone-manipulation study. A significant recurrence of PMDD symptoms was defined by a weekly average DRF score of greater than three (moderate severity) in irritability, anxiety or sadness.^40, 41^ Asymptomatic controls had no affective symptoms during the 6 months (that is, no weekly average score ⩾2). At each clinic visit, all women completed the Rating for Premenstrual Tension self-ratings,^42^ a self-report rating that measures mood, behavior and physical symptoms on a 36-point scale, with scores ⩾ 10 consistent with PMDD symptoms.

### Blood samples

Serum was obtained and stored at −80 °C for metabolomics analysis after at least 8 weeks of GnRH agonist-induced ovarian suppression (hypogonadal state and PMDD symptoms in remission), after 2–3 weeks of estradiol and after 2–3 weeks of progesterone addback (when symptoms recur in PMDD but not in control women).

### Selection of steroid metabolites

We tested 49 metabolites with a focus on estradiol, progesterone and pregnenolone biosynthesis (see Table 1 and Supplementary Table S1, and the Kyoto Encyclopedia of Genes and Genomes: http://www.genome.jp/kegg/pathway.html). We selected direct precursors or metabolites of estradiol (estrone, estriol and so on), progesterone (pregnenolone, pregnenolone sulfate (3b-hydroxy-5-pregnen-20-one-3-SO_4_), allopregnanolone (3a-hydroxy-5a-pregnan-20-one)) and testosterone (androstenedione, dehydroepiandrosterone (DHEA)) and metabolites with neuroactive potential.^43, 44^ Of particular interest were steroids with direct actions at the GABA_A_ receptor, including pregnenolone and DHEA (direct or indirect positive modulators) or their sulfated metabolites, pregnenolone sulfate and dehydroepiandrosterone-SO_4_ (DHEAS; direct or indirect negative modulators).^43, 44, 45^ The sulfation of several neuroactive steroids converts agonist actions at GABA_A_ receptors to antagonist effects, whereas removal of the sulfate group has the opposite effect.^43, 44, 45^

Finally, we measured levels of cortexolone, cortisol, corticosterone and cortexone to evaluate possible alternate pathways of steroid metabolism (analogous to the diversion of cholesterol precursors from corticosteroids toward sex steroid synthesis in congenital adrenal hyperplasia^46, 47^).

### Sample preparation and analysis

Serum samples were extracted and subjected to ultraperformance liquid chromatography tandem mass spectrometry analysis for measurement of neurosteroids in serum samples as described previously^27^ (see Supplementary Methods for detail).

### Statistical analyses

#### Demographic and clinical differences across diagnostic groups

To compare demographic and clinical characteristics of women with PMDD and control women (that is, age, body mass index (BMI), days of exposure to Lupron, E2 and P4, storage times,^48, 49^ parity and race), we used Student’s *t*-tests and *χ*^2^-tests (or Fisher’s exact test when appropriate), with a significance threshold of *P*<0.05, uncorrected for multiple comparisons. We employed analysis of variance with repeated measures (ANOVA-R) to examine potential differences in serum levels of estradiol and progesterone with diagnosis (that is, PMDD and control women) as a between-group factor and hormone condition (that is, Lupron alone, estradiol addback and progesterone addback) as the within-subject factor. Finally, we also used ANOVA-Rs to examine differences in symptom severity across hormone conditions or between diagnoses.

#### Metabolomic differences across diagnostic groups

We did not use metabolite data with greater than 40% of values below the individual assay’s limits of detectability (LOD) in any of the two diagnostic groups or in any of the three hormone conditions. These non-detectable metabolites were distributed across both diagnostic groups and all hormone conditions. Thus, metabolites meeting this criterion in any hormone condition were not included in analyses examining the change in the metabolite level from Lupron to E2 or Lupron to P4, or the differences across diagnoses in basal metabolite levels within each hormone condition.

To analyze differences in metabolite concentrations between the women with PMDD and control women, we used the open-source statistical software, R (R Foundation for Statistical Computing, Vienna, Austria; ISBN 3-900051-07-0 http://www.R-project.org/).

Women were first stratified by diagnostic group (PMDD and controls), and within each diagnostic group we tested for significant metabolite differences between each of the three hormone conditions using a non-parametric, paired Wilcoxon rank-sum test. Subsequently, changes in metabolite levels between PMDD and control women (that is, diagnosis-related differences in the 'delta' metabolite level) from Lupron to E2 and from Lupron to P4 were tested using an unpaired Wilcoxon rank-sum test. All results were corrected for multiple comparisons using a false-discovery rate approach (*q*<0.2).^50, 51^

## Results

### Sample characteristics

Demographics and clinical characteristics of women with PMDD and control women are listed in Table 2. Women with PMDD were significantly older and had higher BMI compared with control women (both comparisons *P*<0.05). There were no between-group differences in racial distribution, parity, the durations of exposure to Lupron, E2 or P4 prior to obtaining the serum samples, storage times or race (*P*>0.05 for all comparisons). Only one woman with PMDD had a past major depression, the rest had no past Axis I psychiatric illness. Ten women with PMDD experienced symptom recurrence only after exposure to the P4, four women only after E2 exposure and one under both P4 and E2 exposures. There was a significant diagnosis-by-hormone interaction in severity scores on the Premenstrual Tension-rater (*P*=0.02) reflecting significantly greater symptom severity in PMDD during addback compared with Lupron alone and compared with control women during addback of E2 or P4 treatment.

### Levels of serum estradiol and progesterone during Lupron alone and after each hormonal addback condition

There were no significant effects of diagnosis or a diagnosis-by-hormone condition interaction for levels of either estradiol or progesterone. All women (PMDD and control) treated with E2 showed significant increases in serum estradiol after E2 treatment compared with Lupron, and significant increases in serum progesterone levels after P4 treatment (Table 3).

### Comparisons of basal steroid metabolite levels between women with PMDD and controls

There were no differences in absolute steroid metabolite levels between women with PMDD and controls in the Lupron, E2 or P4 addback conditions (Table 3).

### Differences in steroid metabolite levels from Lupron to E2 or Lupron to P4 treatment

#### Changes in steroid metabolite levels observed in both PMDD and Controls

Compared with the Lupron condition, treatment with E2 (Table 4) resulted in significant increases, in both PMDD and control women, in levels of estrone-SO_4_ and estradiol-3-SO_4_ levels (Table 4).

Compared with the Lupron condition, replacement of P4 (Table 5) resulted in significant increases, in both PMDD and control women, in serum levels of allopregnanolone and pregnanediol. Serum levels of cortexone also were significantly increased in both groups.

#### Changes in steroid metabolite levels that differed between PMDD and Controls

Only estradiol-3-SO_4_ levels showed a significant diagnostic difference between PMDD and control women after E2 treatment compared with Lupron (Table 4). Women with PMDD had a significantly attenuated (that is, blunted) increase in estradiol-3-sulfate after E2 compared with control women. Other E2-related changes in steroid metabolite levels did not differ between PMDD and control women. Despite the absence of other significant between-group differences, significant within-group differences between Lupron and hormone-replaced were observed in one group but not in the other. Within-group differences in E2-treated women with PMDD included significant decreases in estrone, pregnenolone sulfate (3b-hydroxy-5-pregnen-20-one-3-SO_4_), DHEAS and DHEA levels, compared with no significant change, or a trend toward increased levels of these metabolites in controls (Table 4).

Only DHEAS levels showed a significant diagnosis-related difference between PMDD and control women after P4 treatment (Table 5), with a decrease in serum levels in PMDD and an increase in controls. Notably, none of the four progesterone-related neurosteroid metabolites successfully measured (9-dehydroprogesterone, 3a-hydroxy-5a-pregnan-20-one (allopregnanolone), 17a, 20a-dihydroxyprogesterone and pregnanediol) showed significant diagnostic differences after P4. In particular, the magnitude of the increases in allopregnanolone levels was almost identical in PMDD and controls (Table 5).

Despite the absence of other significant between-group differences, significant within-group differences between Lupron and hormone-replaced conditions were observed in one group but not in the other. Estradiol-3-SO_4_ levels and 2-hydroxyestrone decreased significantly in P4-treated women with PMDD but not in controls. The significant increases in 17a, 20a-dihydroxyprogesterone and androstenedione levels observed in control women were not seen in women with PMDD. Finally, only women with PMDD showed a significant increase in cortexolone levels (Table 5).

## Discussion

We examined the hypothesis that altered metabolic processing of ovarian steroids after E2/P4 exposure would distinguish women with PMDD from matched, asymptomatic controls. The women with PMDD met clinical criteria for PMDD and, of particular relevance, were also confirmed to be behaviorally sensitive to E2/P4.^5^ Controls lacked any significant menstrual cycle or hormone-related behavioral symptoms. Both groups received identical hormone manipulations to standardize exposures. We used a targeted metabolomics platform to detect differences in steroid metabolites in these highly selected clinical phenotypes exposed to identical doses of E2 and P4 while receiving Lupron.

Despite several studies reporting abnormal levels of P4-derived neurosteroids in PMDD, our analysis revealed few significant differences between women with PMDD and controls. Notably, serum levels of the ring A-derived neurosteroid metabolites of P4 were virtually identical in the groups during all hormonal conditions. These similar serum levels also suggest that a larger sample size would be unlikely to reveal significant differences. These findings contrast with several,^16, 19, 20, 21, 22^ but not all, naturalistic studies across the menstrual cycle^17, 18^ and suggest that basal abnormalities of neurosteroid production do not contribute to the pathophysiology of PMDD. Thus, our data confirm, under controlled conditions, the absence of metabolic differences in the formation of 5-alpha-reduced (and 3-beta-hydroxy steroid dehydrogenated) metabolites of P4 in PMDD compared with controls. The neurosteroid hypothesis for PMDD has been very attractive but has been inadequately tested, to date, for the following two reasons: (1) the actions are largely determined by the integrated neurosteroid signal from the profile of metabolites, which, until now, have not been accessible for measure; and (2) samples of women with PMDD are heterogeneous with respect to the critical variable—hormone sensitivity. Both of these obstacles have been overcome in the current study, thus enabling us to answer, albeit negatively, a critical etiological question (and one that is all the more important to address, given recent demonstration of the efficacy of a neurosteroid in another reproductive endocrine-related mood disorder, postpartum depression (PPD;^52, 53^ despite the lack of evidence of abnormal levels of allopregnanolone in PPD). Nonetheless, our findings of otherwise normal P4-derived neurosteroid levels in PMDD are consistent with recent evidence that the luteal-phase increase (that is, change) in neurosteroid levels—not basal levels—mediates the onset of symptoms in women with PMDD,^23^ as blockade of 5-alpha reductase activity mitigated the PMDD symptom onset. Finally, it is possible that our inability to demonstrate abnormalities of neurosteroid levels or profiles may reflect our selection of a distinct, homogeneous subphenotype—women with documented ovarian hormone-induced behavioral sensitivity—and therefore may not generalize to the larger and more heterogeneous population of women with PMDD.

Our findings suggest that after a short-term exposure, women with PMDD process ovarian steroids differently from control women. Specifically, women with PMDD had relatively lower serum levels of the sulfated metabolites of the neurosteroids DHEA, pregnenolone and estradiol after exposures to either E2 or P4. Sulfation of hydrophobic steroids reduces steroid binding to its receptor and facilitates the transfer of the steroid from the tissues into the circulation (in favor of excretion).^54^ The relative balance of sulfated and non-sulfated neurosteroid metabolites determines the actions of these compounds at excitatory and inhibitory neuronal systems. Thus, the reduction in sulfated metabolites observed in PMDD could contribute to the development of PMDD symptoms, and, therefore, reflect the presence of a differential processing of the steroid signal. Nonetheless, decreased levels of sulfated neurosteroids would be predicted to reduce glutamatergic activity, promote GABA agonism and, therefore, favor anxiolytic effects instead of the symptom provocation that occurs in the women with PMDD during the addback phase of the study.

The decreased peripheral levels of sulfated neurosteroids in PMDD occurred in the absence of detectable differences in the non-sulfated forms of these same steroids (that is, estradiol, DHEA and pregnenolone). These differences also are not explained by differential hormone exposure as no diagnosis or diagnosis-by-hormone condition effects were observed in peripheral levels of E2/P4, and there were no differences in sulfated steroids during Lupron alone.

The relative balance between sulfated and non-sulfated steroids is maintained by two families of enzymes, the sulfotransferases (SULT) and sulfatases (STS) working in concert with sulfate group donor molecules, 3’-phospho-adenosine-5’-phosphosulfate (PAPS) and its synthetic enzyme PAPS-synthase, as well as organic anion transporter proteins that control influx and efflux into and out of the cell, respectively.^54, 55^ In humans, both abnormalities in the regulation of this system and mutations of enzymes and transporter-protein genes are associated with disease states including behavioral conditions such as autism and attention-deficit disorder.^54^ The presence of lower E2-3-sulfate in PMDD suggests an abnormality within the SULT family of enzymes, as in humans E2-3-sulfate is not a substrate for STS (see http://www.genome.jp/kegg/pathway.html#metabolism (humans)). Similarly, the absence of diagnostic differences in either estrone or its sulfated metabolite suggests that the potential abnormality in the regulation of sulfation is not uniformly present in PMDD.

The regulatory complexity of steroid sulfation prevents a more precise localization of the source for the diagnostic differences observed in this study. Members of the SULT family are differentially localized within the brain, regulated by a wide range of nuclear receptors and steroids (for example, by P4 and therefore could show increased SULT activity in the luteal phase of the menstrual cycle^56^), and exhibit substrate-induced feedback inhibition.^54^ Although there was no clear evidence of an effect of progesterone in this study, the difference in DHEAS levels after P4 addback (that is, decreasing in PMDD and increasing in control women) might reflect differential processing of P4. Alternatively, P4-induced changes in SULT could have been obscured by changes in STS. Nonetheless, the current data seem to contradict any significant luteal phase effect directly related to P4.

Abnormalities of both DHEAS and pregnenolone sulfate have been suggested to contribute to abnormal affective states,^45, 57, 58^ and, therefore, it is possible that the altered profile of sulfated neurosteroids relative to controls directly contributed to PMDD symptom recurrence. Few studies have evaluated blood levels of sulfated DHEA, E2 and pregnenolone during the luteal phase in women with PMDD and controls. One study^17^ reported no significant differences in pregnenolone sulfate levels between women with PMDD and controls, and, in contrast to our findings, higher plasma levels of pregnenolone sulfate were associated with more severe symptoms in PMDD. Similarly, although DHEAS may have potent affective state altering effects in women, no study has observed significant diagnostic differences in DHEAS in women with PMDD. Alternatively, as sulfation is regulated by both estrogen and progesterone receptors (and their cognate ligands), and women with PMDD exhibit altered luteal-phase responsivity to E2/P4 in a range of phenomena,^5, 59, 60, 61, 62^ the differences in sulfated metabolites could reflect further evidence of abnormal steroid signaling in PMDD that is not directly causing symptoms. Finally, as glucocorticoids and other stress-related factors (that is, soluble cytokines) can regulate steroid sulfation,^54^ it is possible that the altered levels of sulfated steroids occur secondary to the effects of stress or the presence of a negative affective state in women with PMDD during E2/P4 addback.

Several limitations in this study deserve mention. It comprised a small sample, so that metabolite analysis did not predict which women with PMDD were progesterone responders (that is emergence of PMDD symptoms on progesterone) versus estrogen responders, or precisely localize sulfation pathway abnormalities in PMDD. Our selection criterion excluded over 50% of metabolites with low levels, thus limiting the number of analyses. The main goal of this study was to examine the potential differences in the metabolic processing of standardized doses of ovarian steroids in women with PMDD and controls. Although potential confounding factors (for example, age, race, parity and BMI) were not a concern for testing metabolite changes within diagnostic groups, because of the paired study design, this was not the case for testing metabolite changes between PMDD and control groups. For these tests, the small sample size did not allow for adequate statistical control for age, race and BMI, and additional research is needed to determine the impact of these factors on metabolite levels in women with PMDD. Women with PMDD and control women did not significantly differ in either racial distribution or parity. Levels of sulfated steroids can decline with age; however, the role of declining age on SULT activity remains unclear with one study describing age-related changes in SULT2A1 expression,^54^ although others report no changes with age.^46, 54, 63, 64^ Similarly, few studies have documented an effect of age on 5-alpha-reductase activity.^65^ Thus, it is unlikely, albeit, possible that the 6-year difference in age between the two groups underlies their differences in E2/P4-induced sulfated steroid levels. Similarly, evidence in humans suggests that at least SULT1E1 is pro-adipogenic, and, therefore, the higher average BMI observed in the women with PMDD would be consistent with increased (not decreased) sulfotransferase activity.^54, 66^ The sample size also limited our ability to examine whether race and ethnicity or parity contributed to the observed differences in hormone levels. Although women with PMDD and controls did not significantly differ in these characteristics, nonetheless, we should include the caveat that the literature does not allow one to conclude that these factors (age, BMI, race and parity) in women significantly regulate the synthetic enzymes relevant to our findings; that is, STS and SULT (differential effects) and 5-alpha reductase (no differential effects). In addition, the design of this study was deliberately constructed to use the GnRH agonist-induced ovarian suppression to create a hormonally uniform hypogonadal 'baseline', a controlled condition permitting better assessment of the effects of a physiologically relevant and standardized ovarian steroid challenge. Our data suggest but do not definitively demonstrate that these inferred alterations in sulfation enzymes are relevant to the onset of PMDD symptoms during the luteal phase of the menstrual cycle when levels of estradiol and progesterone increase. Clearly, these pilot data need to be followed-up in the naturalistic setting during the course of the normal menstrual cycle in a sufficiently large sample of women to control for differences in substrate (that is, estradiol and progesterone secretion) as well as more specific evaluations of SULT and STS enzyme activities. A more comprehensive assessment of those steroid metabolites in which very low levels of several metabolites could not be reliably measured (as the levels were below the limits of detection for the steroid assays employed in this study) is also necessary to pursue these potential diagnostic-related differences in steroid metabolism. Finally, the generalization of our findings to the larger population of women with PMDD is limited by the strict research criteria employed.

This is the first study, to our knowledge, to employ a pharmacometabolomics approach to investigate the pattern of both basal and stimulated levels of steroid metabolites in women with well-characterized PMDD and controls. Notably, the same dose of P4 was not metabolized into P4-derived neurosteroids differently in PMDD and control women. However, we found differences in the sulfation of estradiol, DHEA and pregnenolone in women with PMDD when exposed to either E2 or P4, suggesting that differences in the metabolism of sulfated neurosteroids could be a source of the observed differential steroid sensitivity in PMDD, and, therefore, could contribute to the underlying pathophysiology of this condition.

## Figures and Tables

**Table 1:** Restricted neurosteroid panel

*Androgens*	*Estrogens*	*Progestogens*	*Corticosteroids*
Dehydroepiandrosterone	Estrone	9-Dehydroprogesterone	Cortexolone
DHEAS	Estradiol	Progesterone	Cortisol
Dehydroepiandrosterone glucuronide	Estradiol-3-SO_4_	3a-Hydroxy-5a-pregnan-20-one (allopregnanolone)	Cortexone
Androstenedione	Estrone-3-SO_4_	17a,20a-Dihydroxyprogesterone	Corticosterone
Testosterone	Estrone-3-glucuronide	3b-Hydroxy-5-pregnen-20-one-3-SO_4_ (pregnenolone sulfate)	
	2-hydroxyestrone	Pregnanediol	

Abbreviations: DHEAS, dehydroepiandrosterone-SO_4_; LOD, limit of detectability; SO_4,_ sulfate group.

This table shows the restricted neurosteroid panel, which outlines the steroid metabolites meeting the threshold for inclusion (<40% of values below LOD) in all of the three hormone conditions (total 21 metabolites, out of 49 metabolites tested).

The complete neurosteroid panel (see Supplementary Table S1) included 49 selected steroid hormones that are direct precursors or metabolites of testosterone, estradiol and progesterone, as well as their sulfated derivatives. We also measured corticosteroid levels, in order to rule out decreased activity in the synthetic enzymes for sex steroids leading to a diversion of cholesterol precursors toward corticosteroid synthesis.

**Table 2:** Demographics and clinical characteristics of women with PMDD and control women

	*Control*	*PMDD*
Age (years), mean (s.d.)*	32.9 (8.1)	39.1 (7.3)
BMI (kg/m^2^), mean (s.d.)*	24.2 (3.5)	27.9 (5.6)
Lupron exposure (days), mean (s.d.)	68.9 (4.0)	71.5 (4.6)
Estradiol exposure (days), mean (s.d.)	20.5 (4.2)	21.8 (6.7)
Progesterone exposure (days), mean (s.d.)	21.9 (4.7)	19.9 (4.1)
Storage times (years)	5.7 (1.9)	6.2 (2.4)
		
*Race (numbers of women)*		
White	7	5
Black	3	8
Hispanic	4	1
Asian	0	1
Other	1	0
		
*Parity*		
Parity=0	9	5
Parity=1–5	6	10
		
Past Axis I Psychiatric Illness^#^ (numbers of women)	0	1
		
Symptom trigger (numbers of women)*		
Progesterone	0	10
Estradiol	0	4
Both	0	1
		
PMDD symptom severity (Steiner–Carroll scale: self-report)* mean (s.d.)		
Lupron	0.6 (1.1)	1.8 (1.6)
E2	0.1 (0.3)	2.8 (3.1)
P4	0.3 (0.8)	6.1 (7.1)

Abbreviations: ANOVA-R, analysis of variance with repeated measures; BMI, body mass index; E2, estradiol; P4, progesterone; PMDD, premenstrual dysphoric disorder.

Demographics and clinical information of the sample are shown in this table. Women with PMDD were significantly older, had higher BMI and showed significant differences in PMDD symptom severity compared with controls under all hormone conditions, as well as across hormone conditions (significant comparisons indicated with an asterisk*, *P*<0.05). One woman with PMDD had a past major depression (indicated with a pound sign^#^). There were no between-group differences in the durations of exposure to Lupron, E2 or P4 prior to obtaining the serum samples, storage times, parity or race (*P*>0.05 for all comparisons). Ten women with PMDD experienced PMDD symptom recurrence only after exposure to the P4, 4 women only after E2 exposure only and 1 after both P4 and E2 exposures. There was a significant diagnosis-by-hormone interaction in severity scores on the Premenstrual Tension-rater (F_2, 56_=4.1, *P*=0.02) reflecting significantly greater symptom severity in PMDD during addback compared with Lupron alone and compared with control women during addback of E2 or P4 treatment. ANOVA-R showed no significant effects of diagnosis (PMDD versus control women) or a diagnosis-by-hormone condition interaction between diagnosis and hormone condition for serum levels of either E2 (diagnosis: F_1,28_=1.3, *P*=0.3; diagnosis-by-hormone interaction: F_2,56_=0.5, *P*=0.6) or P4 (diagnosis: F_1,28_=0.03, *P*=0.9; diagnosis-by-hormone interaction: F_2,56_=0.04, *P*=0.9).

As outlined in Tables 3, 4, 5, all women (PMDD and control) treated with E2, showed a significant increase in estradiol with E2 treatment (PMDD: median change (Median)=72 pg/ml; *q*=0.056; control: Median=61 pg/ml; *q*=0.014), and a significant increase in progesterone with P4 treatment (PMDD: Median=7850 pg/mL; *q*=0.001; control: Median=8530 pg/ml; *q*=0.001), relative to the Lupron condition.

**Table 3:** Serum levels of steroid metabolites (pg ml^−1^) in PMDD (*n*=15) and controls (*n*=15) during three hormone conditions—mean (s.d.)

*Treatment; group*	*Lupron alone*		*Estradiol*		*Progesterone*	
	*NC*	*PMD*	*NC*	*PMD*	*NC*	*PMD*
DHEA	1597.5 (2197.5)	1379.5 (1104.3)	1389.1 (1144.9)	815.8 (774.2)	1947.9 (1766.4)	1127.3 (666.0)
DHEAS	977 766.1 (432 417.6)	765 185.6 (485 557.4)	1 101 474.9 (800 163.7)	511 647.2 (321 591.3)	1 231 969.7 (723 437.8)	553 833.9 (339 282.8)
DHEA-Glu	42 015.6 (16 337.3)	34 679.2 (24 736.1)	47 252.7 (25 905.6)	24 304.7 (22 416.4)	47 073.3 (24 028.9)	28 650.9 (21 361.5)
9-Dehydroprogesterone	2535.9 (2141.0)	2732.3 (2133.7)	2052.9 (1507.0)	1570.8 (1588.6)	2288.0 (1273.9)	1748.0 (1297.8)
7a-Hydroxyandrostenediol	1912.3 (1878.5)	2858.8 (6265.4)	NA	NA	1163.7 (1025.7)	1730.6 (2602.7)
Progesterone	67.9 (112.3)	56.7 (100.0)	38.6 (32.0)	64.0 (54.1)	8409.2 (3052.7)	8202.8 (3035.3)
Cortexolone	79.8 (123.9)	105.4 (93.9)	65.6 (82.7)	163.9 (205.7)	86.8 (117.7)	171.7 (142.5)
Cortisol	12 000.2 (10 545.0)	17 695.5 (11 174.2)	11 048.2 (10 547.3)	14 754.7 (12 930.9)	18 922.8 (13 818.5)	21 248.8 (16 876.5)
Cortexone	18.3 (25.9)	31.6 (19.1)	19.9 (22.5)	28.8 (31.0)	49.9 (45.1)	68.3 (51.1)
Corticosterone	614.9 (766.8)	615.5 (668.8)	423.3 (634.5)	657.6 (1170.6)	504.7 (376.2)	911.9 (1390.9)
Androstenedione	383.0 (205.1)	406.1 (264.2)	431.0 (249.8)	343.5 (345.7)	518.3 (253.0)	314.0 (265.5)
Testosterone	110.2 (90.8)	94.8 (85.9)	117.6 (145.7)	67.1 (63.7)	88.6 (69.7)	69.8 (82.2)
Estrone	333.4 (301.8)	405.0 (505.6)	438.6 (678.4)	219.0 (118.1)	264.3 (253.9)	305.4 (180.8)
Estradiol	67.4 (82.3)	65.4 (65.6)	156.1 (90.3)	282.2 (567.5)	35.8 (44.8)	86.5 (134.6)
Estradiol-3-SO_4_	39.2 (23.9)	70.2 (52.1)	179.1 (82.0)	118.7 (94.7)	37.1 (20.5)	46.4 (29.2)
Estrone-SO_4_	333.8 (118.2)	502.4 (295.6)	2116.6 (1378.0)	1542.1 (1347.9)	349.5 (155.6)	496.9 (375.4)
Estradiol-3-Glu	54.6 (126.9)	31.4 (35.0)	40.3 (44.9)	23.0 (33.1)	NA	NA
Estrone-3-Glu	835.9 (368.6)	1361.7 (780.6)	1103.4 (551.6)	1201.6 (885.4)	829.4 (506.9)	1050.2 (469.2)
2-Hydroxyestrone	96.0 (150.6)	72.4 (90.8)	233.8 (646.8)	49.6 (49.1)	68.8 (90.3)	46.5 (42.2)
2-Hydroxyestradiol	NA	NA	NA	NA	NA	NA
2-Methoxy-3 OH-estrone	NA	NA	NA	NA	NA	NA
6b-Hyroxyestradiol	NA	NA	NA	NA	NA	NA
16a-Hydroxyestrone	NA	NA	NA	NA	NA	NA
Estriol	NA	NA	NA	NA	NA	NA
3a-Hydroxy-5a-pregnan-20-one	425.8 (163.6)	367.9 (224.2)	370.4 (134.5)	366.3 (138.3)	744.2 (187.7)	686.1 (268.8)
5a-Dihydroprogesterone	NA	NA	NA	NA	NA	NA
Allopregnanediol	NA	NA	NA	NA	NA	NA
11a-Hydroxy-4-pregnene-3,20-dione	NA	NA	NA	NA	NA	NA
11b-Hydroxy-4-pregnene-3,20-dione	NA	NA	NA	NA	NA	NA
17-Hydroxypregnenolone	NA	NA	NA	NA	NA	NA
17-Hydroxyprogesterone	NA	NA	NA	NA	NA	NA
21-Hydroxypregnanolone	712.2 (715.2)	516.4 (510.6)	NA	NA	NA	NA
21-Hydroxypregnenolone	NA	NA	NA	NA	NA	NA
7a-Hydroxypregnenolone	NA	NA	NA	NA	NA	NA
20a-Hydroxy-5a-pregnan-3-one	NA	NA	NA	NA	NA	NA
20a-Dihydroprogesterone	NA	NA	NA	NA	NA	NA
17a,20a-Dihydroxyprogesterone	90.6 (58.7)	93.8 (65.1)	83.5 (71.5)	73.1 (72.6)	144.5 (67.6)	103.1 (57.9)
3b-Hydroxy-5-pregnen-20-one-3-SO_4_	17 038.1 (12 188.9)	20 008.5 (12 422.2)	17 864.9 (19 268.8)	12 428.5 (11 518.0)	24 646.4 (18 675.7)	21 174.9 (12 905.6)
Eltanolone (pregnanolone)	NA	NA	NA	NA	NA	NA
Pregnanediol	72.2 (129.4)	111.4 (171.2)	NA	NA	503.5 (400.3)	418.7 (318.1)
5b-Dihydroprogesterone	NA	NA	NA	NA	NA	NA
5a-Dihydrotestosterone	NA	NA	NA	NA	NA	NA
17b-Dihydroandrosterone	NA	NA	NA	NA	NA	NA
17b-DihydroEPIandrosterone	NA	NA	NA	NA	NA	NA
7a-Hydroxytestosterone	NA	NA	NA	NA	NA	NA
7a-Hydroxyandrostenedione	190.3 (194.1)	284.1 (244.1)	NA	NA	209.2 (322.9)	363.5 (531.6)
27-Hydroxycholesterol	NA	NA	NA	NA	NA	NA
24-Hydroxycholesterol	NA	NA	NA	NA	NA	NA

Abbreviations: DHEA, dehydroepiandrosterone; DHEAS, dehydroepiandrosterone-SO4; NA, not applicable since values of steroid metabolite was below limits of detectability of the assay; NC, negative control; PMDD, premenstrual dysphoric disorder.

The unit of measurement for all steroid metabolites is pg ml^−1^.

**Table 4:** Changes in steroid metabolites during ovarian steroid replacement of E2

*Metabolite*	*Median Δ*		P-*value*		Q*-value*	
	*PMDD*	*NC*	*PMDD*	*NC*	*PMDD*	*NC*
Estrone-SO_4_	888	1425	0.002	1.22E−04	0.042	0.001
Estradiol	72	61	0.005	0.002	0.056	0.014
Estrone	−73	0	0.012	0.561	0.065^#^	0.978
DHEAS	−119 657	49 563	0.012	0.720	0.065^#^	0.978
3b-Hydroxy-5-pregnen-20-one-3-SO_4_	−6176	959	0.018	0.847	0.076^#^	0.978
DHEA	−324	311	0.028	0.890	0.098^#^	0.978
Estradiol-3-SO_4_*	47	140	0.055	6.10E−05	0.166	0.001

Abbreviations: DHEA, dehydroepiandrosterone; DHEAS, dehydroepiandrosterone-SO_4_; E2, estradiol; Median Δ, median change; NC, normal control; PMDD, premenstrual dysphoric disorder.

The unit of measurement for all steroid metabolites is pg ml^−1^.

This table shows the significant changes in steroid levels in women with PMDD and controls with E2 addback relative to Lupron. Significant differences indicated by *Q*<0.2.

Only estradiol-3-SO_4_ levels showed significant between-group differences following E2 treatment (*P=0.002; *q*=0.035), with a significantly more blunted response to E2 in PMDD compared with control women. Direct comparisons of other E2-related changes in steroid levels between PMDD and control women did not reach significance.

Within diagnostic groups, we observed significant effects of hormone condition in the magnitude of the change in steroid metabolite levels from Lupron to E2 in PMDD but not controls, as follows: women with PMDD showed significant decreases in serum levels of estrone (median Δ: PMDD=−73 pg ml^−1^ (decreased), ^#^*q*=0.065; controls=0 pg ml^−1^, *q*=0.978), pregnenolone sulfate (3b-hydroxy-5-pregnen-20-one-3-SO_4_; median Δ: PMDD=−6176 pg ml^−1^(decreased), ^#^*q*=0.076; controls=959 pg ml^−1^, *q*=0.978), DHEAS (median Δ: PMDD=−119 657 pg ml^−1^(decreased), ^#^*q*=0.065; controls=49 563 pg ml^−1^, *q*=0.978) and DHEA (median Δ: PMDD=−324pg ml^−1^, ^#^*q*=0.098 (decreased); controls=311 pg ml^−1^, *q*=0.978).

**Table 5:** Changes in steroid metabolites during ovarian steroid replacement of P4

*Metabolite*	*Median Δ*		P*-value*		Q*-value*	
	*PMDD*	*NC*	*PMDD*	*NC*	*PMDD*	*NC*
Progesterone	7850	8530	6.10E−05	6.10E−05	0.001	0.001
3a-Hydroxy-5a-pregnan-20-one	344	318	1.22E−04	6.10E−05	0.001	0.001
Pregnanediol	342	506	0.010	0.005	0.077	0.030
DHEAS*	−91 935	159 968	0.018	0.048	0.104	0.157
Cortexone	31	31	0.026	0.041	0.118	0.157
2-Hydroxyestrone	−5.4	−10.7	0.060	0.135	0.183^#^	0.387
Estradiol-3-SO_4_	−26	−12	0.064	0.649	0.183^#^	0.727
Cortexolone	69	0	0.064	0.727	0.183^#^	0.727
Androstenedione	−101	123	0.252	0.035	0.447	0.157^#^
17a,20a-Dihydroxyprogesterone	13	40	0.599	0.002	0.726	0.015^#^

Abbreviations: DHEA, dehydroepiandrosterone; Median Δ, median change; NC, normal control; P4, progesterone; PMDD, premenstrual dysphoric disorder.

The unit of measurement for all steroid metabolites is pg ml^−1^.

This table shows the significant changes in steroid levels in women with PMDD and controls with P4 addback relative to Lupron. Significant differences indicated by *Q*<0.2.

Only DHEAS levels showed significant between-group differences following P4 treatment (*P=0.003; *q*=0.073), decreasing in women with PMDD treated with P4 but increasing in control women subjected to the same hormonal treatment. Direct comparisons of other P4-related changes in steroid levels between PMDD and control women did not reach significance.

Within diagnostic groups, we observed trend effects of hormone condition in the magnitude of the change in steroid metabolite levels from Lupron to P4 in PMDD but not controls, as follows: 2-hydroxyestrone (median Δ: PMDD=−5.4 pg ml^−1^ (decreased), ^#^*q*=0.183; controls=−10.7 pg ml^−1^, *q*=0.387), estradiol-3-SO_4_ (median Δ: PMDD=−26 pg ml^−1^(decreased), ^#^*q*=0.183; controls=−12 pg ml^−1^, *q*=0.727) and cortexolone (median Δ: PMDD=69 pg ml^−1^, ^#^*q*=0.183; controls=0 pg ml^−1^, *q*=0.727). In addition, we also observed significant effects of hormone condition in the magnitude of the change in steroid metabolite levels from Lupron to P4 in controls but not in PMDD, as follows: 17a, 20a-dihydroxyprogesterone (median Δ: PMDD=13 pg ml^−1^, *q*=0.726; controls=40 pg ml^−1^, ^#^*q*=0.015) and androstenedione (median Δ: PMDD=−101 pg ml^−1^(decreased), *q*=0.447; controls=123 pg ml^−1^, ^#^*q*=0.157).
