# Supplementary material for: The steroid metabolome in women with premenstrual dysphoric disorder during GnRH agonist-induced ovarian suppression: effects of estradiol and progesterone addback
Source: Transl Psychiatry. 2017 Aug 8;7(8):e1193–. doi: 10.1038/tp.2017.146 (PMC5611719; doi:10.1038/tp.2017.146)
Supplement: Supplementary Material [file tp2017146x1.docx]

Supplementary Table 1: List of measured metabolites, molecular weight (MW), retention time (RT), limit of detectability (LOD).

| **Metabolite** | **MW** | **RT (min)** | **LOD (pmole)** | **Metabolite** | **MW** | **RT (min)** | **LOD (pmole)** |
| --- | --- | --- | --- | --- | --- | --- | --- |
| **Pregnenolone** | 316.48 | 8.3 | 0.1106 | **3a-Hydroxy-5a-pregnan-20-one** | 318.49 | 9.96 | 0.0110 |
| **DHEA** | 288.42 | 6.19 | 0.6068 | **5a-Dihydroprogesterone** | 316.48 | 10.06 | 0.2212 |
| **DHEAS** | 368.48 | 3.34 | 0.0009 | **Allopregnanediol** | 320.51 | 8.01 | 0.2184 |
| **DHEA-Glu** | 464.55 | 7.26 | 0.1466 | **11a-Hydroxy-4-pregnene-3,20-dione** | 330.46 | 6.34 | 0.0106 |
| **9-Dehydroprogesterone** | 312.45 | 8.82 | 0.0080 | **11b-Hydroxy-4-pregnene-3,20-dione** | 330.46 | 7.19 | 0.0106 |
| **7a-Hydroxyandrostenediol** | 306.44 | 8.14 | 0.0979 | **17-Hydroxypregnenolone** | 332.48 | 7.11 | 0.2105 |
| **Progesterone** | 314.46 | 9.09 | 0.00002 | **17-Hydroxyprogesterone** | 330.46 | 7.5 | 0.0002 |
| **Cortexolone** | 346.46 | 5.87 | 0.0505 | **21-Hydroxypregnanolone** | 334.49 | 8.14 | 1.0464 |
| **cortisol** | 362.46 | 4.7 | 0.0483 | **21-Hydroxypregnenolone** | 332.48 | 6.86 | 0.5263 |
| **Cortexone** | 330.46 | 7.08 | 0.0011 | **7a-Hydroxypregnenolone** | 332.5 | 5.82 | 0.1053 |
| **Corticosterone** | 346.46 | 5.71 | 0.0051 | **20a-Hydroxy-5a-pregnan-3-one** | 304.42 | 9.25 | 0.0575 |
| **Androstenedione** | 286.41 | 7.33 | 0.0012 | **20a-Dihydroprogesterone** | 318.49 | 6.96 | 0.1099 |
| **Testosterone** | 288.42 | 6.83 | 0.0006 | **17a,20a-Dihydroxyprogesterone** | 316.48 | 6.61 | 0.0011 |
| **Estrone** | 270.37 | 4.67 | 0.0006 | **3b-Hydroxy-5-pregnen-20-one-3-SO_4_** | 396.54 | 3.72 | 0.0009 |
| **Estradiol** | 272.38 | 4.63 | 0.0013 | **Eltanolone** | 396.54 | 9.67 | 0.0002 |
| **Estradiol-3-SO_4_** | 352.45 | 3.04 | 0.0020 | **Pregnanediol** | 320.51 | 9.21 | 0.0218 |
| **Estrone-SO_4_** | 372.36 | 2.99 | 0.0009 | **5b-Dihydroprogesterone** | 316.48 | 10.06 | 0.2212 |
| **Estradiol-3-Glu** | 470.49 | 2.71 | 0.0074 | **5a-Dihydrotestosterone** | 290.44 | 7.85 | 0.2410 |
| **30_Estrone-3-Glu** | 446.49 | 2.77 | 0.0078 | **17b-Dihydroandrosterone** | 292.46 | 7.85 | 0.0598 |
| **2-Hydroxystrone** | 286.37 | 4.18 | 0.0012 | **17b-DihydroEPIandrosterone** | 292.46 | 6.94 | 0.0598 |
| **2-Hydroxyestradiol** | 288.38 | 4.11 | 0.0061 | **7a-Hydroxytestosterone** | 304.42 | 4.29 | 0.0230 |
| **2-Methoxy-3 OH-estrone** | 300.39 | 4.78 | 0.0012 | **7a-Hydroxyandrostenedione** | 302.41 | 4.9 | 0.0231 |
| **6b-Hyroxyestradiol** | 288.38 | 3.44 | 0.0024 | **27-Hydroxycholesterol** | 402.65 | 10.0 | 0.4346 |
| **16a-Hydroxyestrone** | 286.37 | 3.98 | 0.0024 | **24-Hydroxycholesterol** | 402.7 | 10.1 | 0.4346 |
| **Estriol** | 288.38 | 6.82 | 0.3470 |  |  |  |  |

Legend to supplementary table 1:

This table shows the complete neurosteroid panel (total 49 metabolites). These steroid metabolites cover multiple components of the steroidogenesis pathway with emphasis on those pathways involved in androgen, estrogen and progestin synthesis (see the Kyoto Encyclopedia of Genes and Genomes [KEGG]: http://www.genome.jp/kegg/pathway.html).

Metabolites included in the analyses (<40% of values below LOD in all of the three hormone conditions) are indicated by white boxes (total 21 metabolites, also see **Table 1**). Excluded metabolites (>=40% of values below LOD in any of the three hormone conditions) are shaded in grey (total 28 metabolites).

**Supplementary Methods:**

**Sample preparation and analysis:**

Serum samples were extracted and subjected to ultra-performance liquid chromatography tandem mass spectrometry (UPLC/MS-MS) analysis for measurement of neurosteroids in serum samples as described previously (27).

UPLC analyses were carried out using a Waters Acquity UPLC system connected with the high performance Xevo-TQ mass spectrometer. Analytical separations on the UPLC system were conducted using an Acquity UPLC HSS T3 1.8 µ column (1 x 150 mm) at a flow rate of 0.15 ml/min and BEH C18 1.7 µ column (2 x 50 mm) at flow rate 0.2ml/min. For HSS T3 column, the gradient was started with 100% A (0.1% formic acid in H_2_O) and 0% B (0.1% formic acid in CH_3_CN), after 0.1min changed to 80% A over 1 min, and then 45% A over 5min, followed by 20% A in 2min. Finally, over 0.5 min, it was changed to 0% A, then after 13 min it was changed to the original 100% A over 1 min, resulting in a total separation time of 13 min. For C18 column, the gradient was started with 100% A (0.1% formic acid in H_2_O) and 0% B (0.1% formic acid in CH_3_OH), after 0.1min changed to 80% A over 1 min, and then 45% A over 2 min, followed by 20% A in 2min. Finally, over 1 min, it was changed to 0% A, then after 7 min it was changed to the original 100% A over 2 min, resulting in a total separation time of 7 min. The elutions from the UPLC column were introduced to the Xevo-TQ mass spectrometer. All MS experiments were performed by using electrospray ionization (ESI) in both positive ion (PI) and negative ion (NI) mode, with an ESI-MS capillary voltage of 3.0 kV, an extractor cone voltage of 3 V, and a detector voltage of 650 V. The following MS conditions were used: desolvation gas at 600 l/h, desolvation temperature at 350 ºC and source temperature 150º. Pure standards of all targeted neurosteroids were used to optimize the UPLC-MS/MS conditions prior to analysis and performing calibration curves. Elutions from the UPLC column were analyzed in the MRM mode, and resulting data was processed by using TargetLynx 4.1 software (Waters).

Reference standards were run before the first sample, in the middle of run and after the last sample to prevent errors due to matrix effect and day-to-day instrument variations. In addition, after the initial standard and before the first sample, two spiked samples were run to calibrate for the drift in the retention time of all neurosteroids due to the matrix effect. After standard and spiked sample runs several blanks were injected to wash the injector and avoid carry over effect.

**Chemicals:**

Reference standards (Supplementary Table S1) # 1 through 49 were purchased from Steraloids (Newport, RI). All solvents were HPLC grade, and all other chemicals used were of the highest grade available. Stock neurosteroid standard mixture was prepared by mixing 5 μl of 1mg/ml solution of each steroid and adjusting final volume to 1ml by using methanol. All the stock solutions were stored at -80^o^C.
